# Supplementary material for: Generation of TRIM28 Knockout K562 Cells by CRISPR/Cas9 Genome Editing and Characterization of TRIM28-Regulated Gene Expression in Cell Proliferation and Hemoglobin Beta Subunits
Source: Int J Mol Sci. 2022 Jun 20;23(12):6839. doi: 10.3390/ijms23126839 (PMC9224613; doi:10.3390/ijms23126839)
Supplement: Supplementary file 1 [file ijms-23-06839-s001.zip › ijms-1727131-supplementary.pdf]

Supplemental results

Deep sequencing analysis of on-target and off-target sites

Amplicon template preparation

Barcoded amplicons were generated by a two-step PCR. The first round PCR was performed by using 300ng gDNA of each clone as template, KAPA HiFi HotStart ReadyMix PCR Kit (KK2601), and the internal primers, a combination of universal and target-specific sequence with 5’ AmMC6 (5’NH4 – C6) modification. The thermocycler setting consisted of 30 cycles of 98 °C for 20 sec, specific annealing temperature (Tm: 65 – 72 °C) for 15 sec and 72°C for 2 min. Second round PCR was using universal tag as the primer pair incorporating the barcode, purified first-round-PCR-products mixed with equal molar, and KAPA HiFi HotStart ReadyMix PCR Kit performed by High Throughput Genomics Core at Biodiversity Research Center, Academia Sinica.

Data analysis

Bioinformatic pipeline for read preprocessing, sequence mapping, and structural variant calling. Sequencing data demultiplex with PacBio barcode via CCS with barcoding (SMRT link 5.0.1 version). Mapping with reference of on-target and top 3 predicted off-target of each sgRNA by NGMLR, and sorting with SMRT tools. Structural variant calling with the pipeline via Sniffles and scripts (Sedlazeck et al., 2018).

Sedlazeck, F.J.; Rescheneder, P.; Smolka, M.; Fang, H.; Nattestad, M.; von Haeseler, A.; Schatz, M.C. Accurate detection of complex structural variations using single-molecule sequencing. *Nat. Methods* **2018**, *15*, 461–468. https://doi.org/10.1038/s41592-018-0001-7.

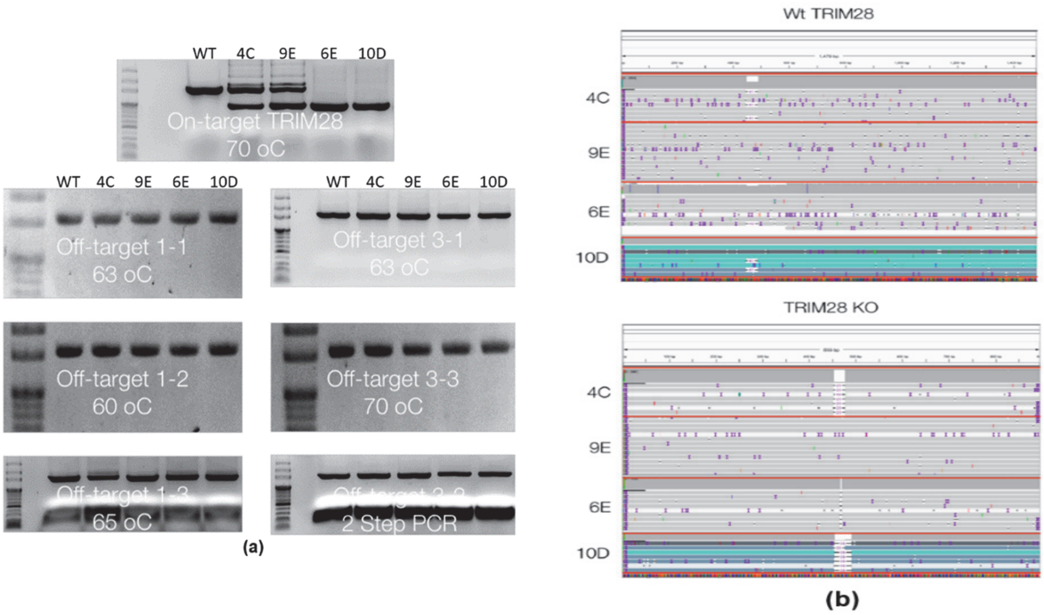

Figure S1. Deep sequencing of amplicon templates (a) PCR across on-target or off-target site to generate amplicon templates in WT, 4C, 9E, 6E and 10D clones for multiplex targeting deep sequencing using KAPA HiFi HotStart Ready Mix PCR kit. Tm of each primer set as labeled. (b) Sequence variation of TRIM28 in each KO clones. Upper panel: 1500 bp wild-type TRIM28 DNA fragment; lower panel: 900 bp mutated TRIM28 DNA fragment.

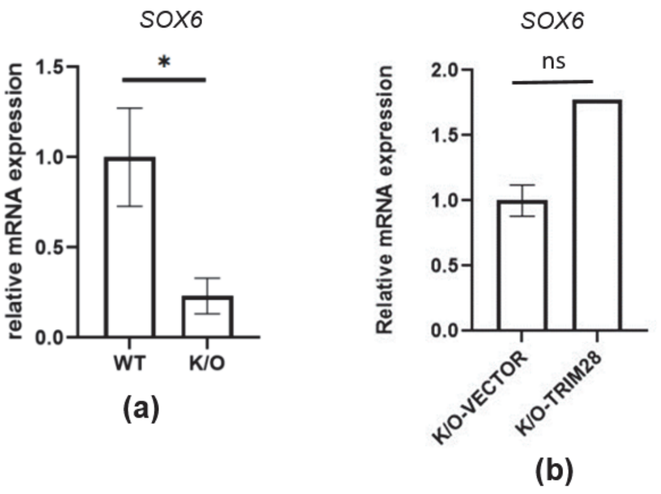

Figure S2. SOX6 is downregulated in *TRIM28* KO K562 cells. RT-qPCR analysis of *SOX6* mRNA expression in WT and *TRIM28* complete-KO K562 cells (a), and in *TRIM28* complete-KO cells transfected with cloning vector or Trim28 (b). The significance was labeled as *p<0.05, or ns (non-significant difference).

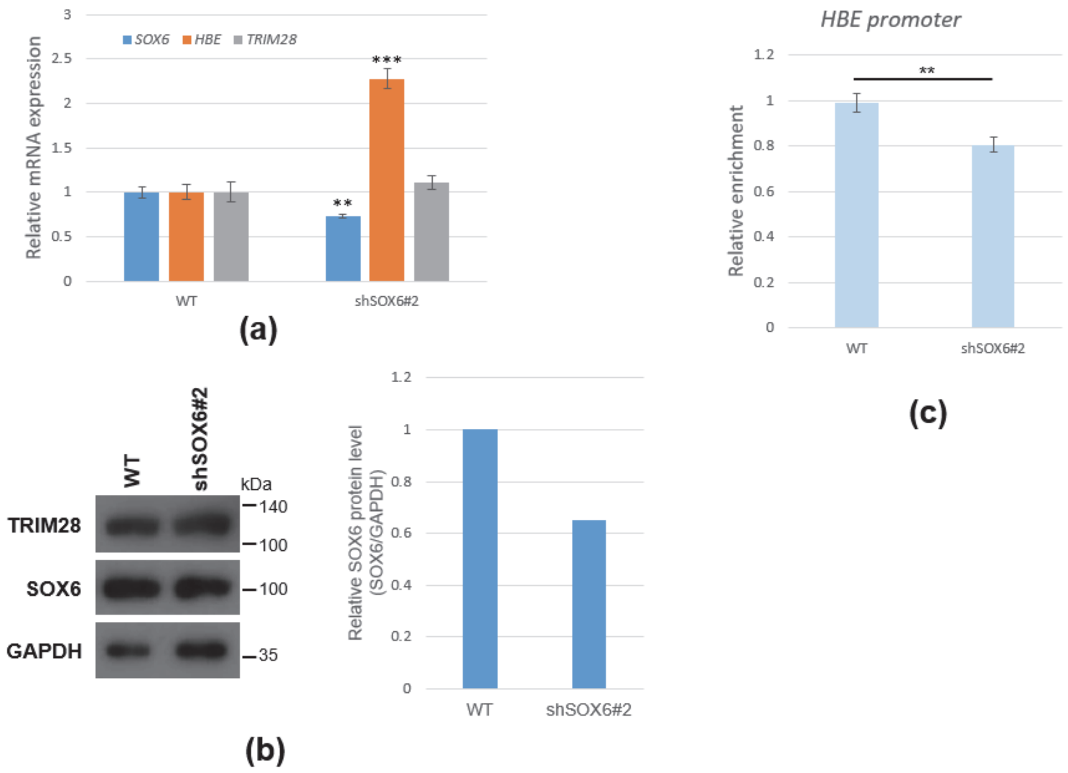

Figure S3. Knockdown of SOX6 decreases TRIM28 enrichment on *HBE* promoter in ChIP assay. (a) The mRNA expression levels of *SOX6*, *HBE* and *TRIM28* in SOX6 knockdown K562 cells. Lentiviral vector-mediated shRNA technology was used to knock down SOX6. The shRNA construct (TRCN0000430184) was obtained from the National RNAi Core Facility at the Academia Sinica, Taipei, Taiwan. Viruses were produced by using calcium phosphate precipitation transfection. 14 μg of pPGK-GFP or pLKO.1-shLuc or shRNA constructs with 14 μg of pCMVΔR8.91 and 2 μg of pMD.G were co-transfected in early subculture of 293T cells on 10 cm dishes. After eight hours, replace the medium with K562 medium to collect virus. Then K562 cells in 6-well plate were infected with the viral supernatants in the presence of 8 μg/ml of polybrene for 48 hours and further selected with 3 μg/ml puromycin (Sigma-Aldrich, St. Louis, MO, USA) for one week. RNAs were isolated from WT and SOX6 knockdown (shSOX6#2) cells for RT-qPCR analysis with primers of SOX6, HBE, TRIM28 and internal control beta-actin. The results were analyzed by the $2^{-\Delta\Delta Ct}$ relative quantitation method. (b) SOX6 protein level in SOX6 knockdown K562 cells. The whole cell extracts were isolated from WT and shSOX6#2 cells and western blotting with anti-TRIM28, anti-SOX6, and anti-GAPDH. The SOX6 protein level was quantitated by iBright imager (Thermo Fisher) using GAPDH as a loading control. (c) ChIP assay. The WT and shSOX6#2 cells were fixed by formaldehyde and IPed with anti-TRIM28. The precipitated genomic DNA was qPCR analyzed with *HBE* promoter primers. The significance was labeled as **p<0.01, or ***p<0.001.

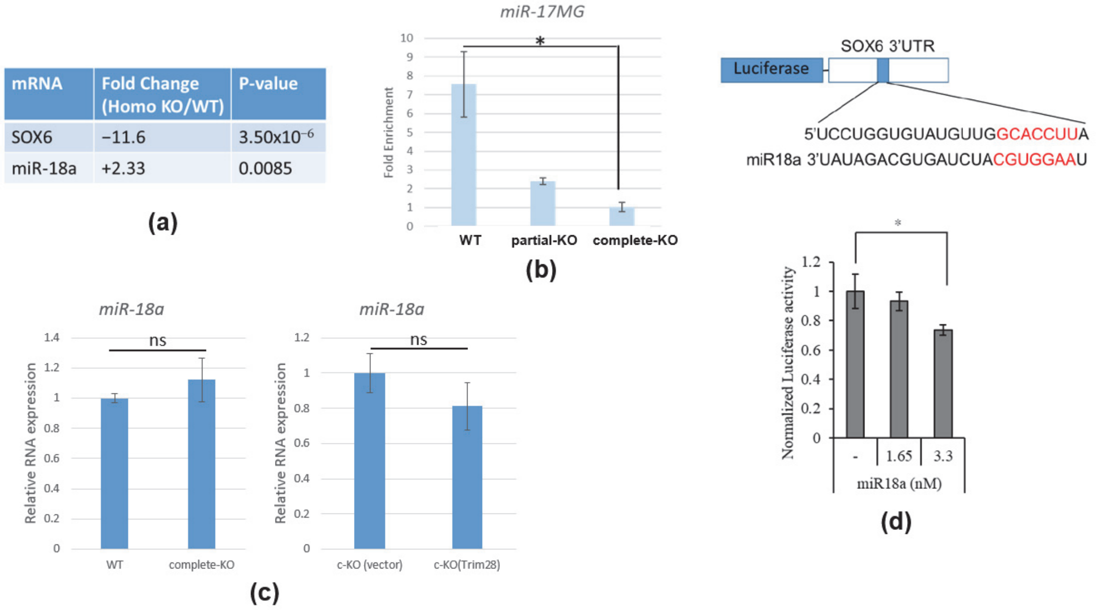

Figure S4. *SOX6* is downregulated by miR-18a in *TRIM28* KO K562 cells. (a) The *SOX6* and *miR-18a* expression in microarray analysis. (b) ChIP assay. The WT, partial-KO, and complete-KO cells were fixed by formaldehyde and IPed with anti-TRIM28. The precipitated genomic DNA was qPCR analyzed with *miR-18a* gene cluster promoter *miR-17MG* primers (forward, 5′-GCTTCAAAATTCATTTGGGATT-3′; reverse, 5′-AGGATTTTGGAAGACGCAAA-3′). (c) miR-18a expression was slightly increased in *TRIM28* complete-KO K562 cells (left panel), and decreased in complete-KO K562 cells overexpressed Trim28 (right panel). The statistic analysis showed no significance. (d) Luciferase assay. The SOX6 3′UTR was PCR cloned with primers (forward, 5′-GTTGTGCAGTATTGACGTGAG-3′; reverse, 5′-CAACAGCAGTACAAAAGATGG-3′), and ligated to 3′ of luciferase gene. HEK293T cells were transfected with SOX6 3′UTR-containing luciferase reporter and miR-18a (Thermo Fisher Scientific). The results were shown after normalized with internal Renilla luciferase activity. The significance was labeled as *p<0.05, or ns (non-significant difference).

Table S1 The target sequence of Cas9-induced site-specific double-strand DNA breaks on TRIM28

	Target sequence	Genomic locus
TRIM28-sgRNA1	GGTTCTCAGCTCGTAAGTGT	Ch.19:+58547702~+58547721
TRIM28-sgRNA2	AGACACCTGGCGGATTCTAG	Ch.19:+58547807~+58547788
TRIM28-sgRNA3	AGATCTACTTCCAGCTGCAC	Ch.19: +58548292~+58548311
TRIM28-sgRNA4	TGGGGACCCACCAAAGGCCT	Ch.19:+58548419~+58548400

Table S2 Internal primers for amplicon template preparation		
TRIM28 On-target	F	5' AmMC6- GCAGTCGAACATGTAGCTGACTCAGGTCAC-CTCTACATCTTCCCAATAAATGGCCCAGTG
	R	5' AmMC6- TGGATCACTTGTGCAAGCATCACATCGTAG-GTGAACAAAGCAGAACCCTCTGCCTCAGT
TRIM28 -sgRNA1 Off-target 1 (ATP9B)	F	5' AmMC6- GCAGTCGAACATGTAGCTGACTCAGGTCAC-GAGCTTACATACTAGGAAACCTTGGGATGC
	R	5' AmMC6- TGGATCACTTGTGCAAGCATCACATCGTAG-CTACAACTCTGGAATTCCATGCCTGTGTTA
TRIM28 -sgRNA1 Off-target 2 (PPP2R5C)	F	5' AmMC6- GCAGTCGAACATGTAGCTGACTCAGGTCAC-CTGGGTAGTGTGGGGAAATAAAAGAACACA
	R	5' AmMC6- TGGATCACTTGTGCAAGCATCACATCGTAG-AGCATCTATGATATCTGAGAGAGGAGGCAC
TRIM28 -sgRNA1 Off-target 3 (ADAMTS12)	F	5' AmMC6- GCAGTCGAACATGTAGCTGACTCAGGTCAC-TAGAATTTAGTGGGGAGGAGGGGAGTTTAC
	R	5' AmMC6- TGGATCACTTGTGCAAGCATCACATCGTAG-ACAGCCATTTCAGAAAACTGCACTAGTGTA
TRIM28 -sgRNA3 Off-target 1 (PIK3CD)	F	5' AmMC6-GCAGTCGAACATGTAGCTGACTCAGGTCAC-AGTCGGTTCCTGCTTCATCTTGTTTTACTT
	R	5' AmMC6- TGGATCACTTGTGCAAGCATCACATCGTAG-AGCAATTTCCCCCAATATGAGTGAAACAGA
TRIM28 -sgRNA3 Off-target 2 (SH2B2)	F	5' AmMC6- GCAGTCGAACATGTAGCTGACTCAGGTCAC-TTATTAGCACCTGTAGTCTCCAGAGTTCGG
	R	5' AmMC6- TGGATCACTTGTGCAAGCATCACATCGTAG-GACAGTGTTAAAAGTCTGAGATCTGGAGCC
TRIM28 -sgRNA3 Off-target 3 (KCNIP1)	F	5' AmMC6- GCAGTCGAACATGTAGCTGACTCAGGTCAC-TTAGAAGTCAGGAGAGCAAAATCCACACAC
	R	5' AmMC6- TGGATCACTTGTGCAAGCATCACATCGTAG-ATCTCCCACAGCCCTATTTATGACCTAGAG

Table S3 qPCR primer sequences		
Name	Forward (5'-3')	Reverse (5'-3')
cDNA		
CCND2 (CyclinD2)	GAGAAGCTGTCTCTGATCCGCA	CTTCCAGTTGCGATCATCGACG
CDKN1 (p21)	AGGTGGACCTGGAGACTCTCAG	TCCTCTTGGAGAAGATCAGCCG
MAGEC2	GGCCCTGAGGAAGAACTGAG	TGAGATCCAACAGGCCTTGAC
MAGEA9	CGTTGAGTGTGATGGGGGTG	ATAAAGGGATGGGTAGCAGATGG
HBG	TGGATGATCTCAAGGGCAC	TCAGTGGTATCTGGAGGACA
HBE	GCAAGAAGGTGCTGACTTCC	ACCATCACGTTACCCAGGAG
SOX6	CGGTCTACCTACTGGGATAA	GCTTTTGTTTGGCAGATTGA
TRIM28	AAGGACCATACTGTGCGCTCTAC	ACGTTGCAATAGACAGTACGTTCAC
GAPDH	CAACAGCGACACCCACTCCT	CACCCTGTTGCTGTAGCCAAA
ACTIN	GCACCAGGGCGTGATGG	GCCTCGGTCAGCAGCA
ChIP		
HBE promoter	GCCAGAACTTCGGCAGTAAA	GGCCTGAGAGCTTGCTAGTG
MAGEC2 promoter	AGAGATGTGGAAGGACGGTG	TAAGCCTTGCGGACCTCTTCAC
miR-874 promoter	TACCTGGTTCTCAGACCCCC	CACCAACAATTCATCCGATG
SOX6 promoter	CTGCAGAGCTGAAGTCGATTC	TCGATAGTGCATTTCAACGC
